# Age-related differences in fall migration timing and performance of juvenile and adult Wood Thrushes departing from a breeding site

**DOI:** 10.1186/s40462-025-00556-3

**Published:** 2025-05-06

**Authors:** Brendan P. Boyd, Sue M. Hayes, Anna Agazzi Migotto, Bridget J. M. Stutchbury

**Affiliations:** Department of Biology, York University, Toronto, Canada

**Keywords:** Migration, Motus, Automated telemetry, *Hylocichla mustelina*, Tailwind, Migration performance

## Abstract

Juvenile passerines are expected to have lower migration performance than adults due to their inexperience with long-distance flights and morphological limitations, such as shorter wing length. From 2016 to 2019 we radio-tagged nestling and adult Wood Thrushes (*Hylocichla mustelina*) at a breeding site in southwestern Ontario and used the automated Motus Wildlife Tracking System to test if age class predicts timing of the onset of fall migration (date, time of night), flight speed during the initial migration flight across Lake Erie, and overall pace of migration southward through the eastern United States. We detected 60/117 (51%) adults and 82/119 (69%) juveniles departing the breeding area as they initiated fall migration. Compared with adults, juveniles departed at an earlier date in fall and later time in the evening. When crossing Lake Erie on their first migratory flight juveniles travelled about 25% slower than adults but this was due primarily to adults making better use of tailwinds. When travelling south through the eastern U.S. juveniles had a slower overall migration pace (47.3 ± 5.1km/day) than adults (71.6 ± 4.7km/day). Although we found evidence that juvenile Wood Thrushes have an earlier and slower fall migration than adults, identifying the proximate and ultimate mechanisms remains a challenge. There is no evidence that juvenile Wood Thrushes are inefficient in migration flight or refueling at stopovers, and it is unlikely that the fall migration pace in this species affects their ability to compete for wintering food resources. More tracking studies from breeding sites are needed to understand the ecological factors favouring and biological significance of, age-related differences in migration performance.

**Supplementary Information:**

The online version contains supplementary material available at 10.1186/s40462-025-00556-3.

## Introduction

Every fall, Neotropical-Nearctic migratory songbirds must travel from their temperate breeding grounds to the tropics and juveniles complete this journey for the first time by using information that has been innately programmed (reviewed by Akesson et al. [1], Justen et al. [35]). This long-distance migration imposes extreme physiological demands on individuals who undergo multi-hour flights followed by multi-day stopovers to refuel [72]. Migration is the most dangerous portion of the annual cycle (e.g. Sillett and Holmes [60]) and as such, it is expected to be under high selection pressure [3]. Optimal migration theory hypothesizes that minimizing time spent on migration is important, but this must be balanced with minimizing both energy cost and predation risk (Hedenstrom [30]). Adults that have already successfully migrated at least once learn routes, stopover sites, final destinations and can make navigational adjustments on successive journeys or after experimental displacement (e.g. Thorup et al. [69]). Thus, adults are expected to have better migration performance than juveniles (e.g., timing of departure, orientation, pace, use of wind assistance; Mitchell et al. [43], reviewed by Akesson et al. [1]). Juvenile birds also have physiological factors that can limit migration performance such as shorter wings [2, 39, 49], larger digestive organs, and smaller flight muscles [32, 38]. Ideal tests of superior migration performance in adults would be longitudinal to show that individuals improve their migration performance as they age [59]. However, repeat tracking is difficult in passerines because juveniles that depart from their natal site on fall migration are rarely encountered again and the battery life of appropriately sized remote tracking devices is not sufficient to gather migration data over multiple years for most species.

Tests of the hypothesis that adult passerines have better migration performance than juveniles have often relied on banding and tracking studies at stopover sites. Schmaljohann et al. [57] found that juveniles departed a stopover site later in the night than adults during fall migration but not spring migration. Several studies have found that during fall migration juveniles spend more time at stopover sites than adults (e.g. Yong et al. [73]; Collet and Heim [16]) which could be due to less efficient refueling and thus cause a slower migration pace. However, body mass gain during fall stopover is not necessarily slower for juveniles (e.g. Rguibi-Idrissi et al. [55], Seewagen et al. [58]). At a Gulf Coast stopover, prior to crossing the Gulf of Mexico in fall, juvenile Swainson’s Thrushes (*Catharus ustulatus*) did not have longer stopovers or depart in a different direction than experienced adults (Smolinsky et al. [62]). Interpretation of such results from stopover studies may be complicated by the different breeding/natal site origins of individuals captured at stopover sites and thus differences in time elapsed since migration departure, distance covered, and different environmental conditions encountered along the way. Juveniles at a stopover site far from their natal site may also have already gained sufficient migration experience to keep pace with adults.

A powerful test of the hypothesis that adults will have better performance is to compare migration parameters for adults and juveniles that begin their migration from the same site. McKinnon et al. [39] deployed geolocators on juvenile and adult Wood Thrushes (*Hylocichla mustelina*) at a wintering site in Belize and found that juveniles started their first spring migration about one week later than adults and had a slower pace of spring migration due to more frequent stops as they moved northward through the U.S. after crossing the Gulf of Mexico. However, geolocators are generally not useful for tracking fall migration of juvenile passerines from natal sites because most individuals are never seen again, and so archival data cannot be retrieved. It is possible however, to remotely track juveniles from their natal site using the automated Motus Wildlife Tracking System (Motus), and either by tagging birds on island breeding populations (e.g. Mitchell et al. [43], Crysler et al. [20]) or by deploying radio-tags on adults and nestlings on the breeding territories months before fall migration begins (e.g. Horn et al. [31], Hayes et al. [28, 29]). Studies on the Savannah Sparrow (*Passerculus sandwichensis*) found that juveniles started fall migration 3–4 weeks earlier than adults ([20, 31]). Mitchell et al. [42] found that juvenile Savannah Sparrows leaving Kent Island, New Brunswick, on their first migration flight took longer to cover the same distance across the ocean as adults but this was a result of juveniles departing under less ideal wind conditions rather than reduced flight performance. Juvenile Savannah Sparrows departing their natal sites on Sable Island, 100 km offshore from Nova Scotia, chose shorter overwater crossings than adults, perhaps to reduce the mortality risk during their first migratory flight [20].

The Wood Thrush is a relatively large (~ 50 g) forest songbird that is a long-distance migrant which has been declining throughout most of its range (e.g. Taylor and Stutchbury [67]). Geolocator tracking has shown that Wood Thrushes from this breeding region primarily winter in eastern Central America [63]. We used Motus in eastern North America to detect migrating juvenile and adult Wood Thrushes that had been radio-tagged on their natal/breeding territories in southwestern Ontario. To evaluate the hypothesis that adults have better migratory performance than juveniles, we compared the date of fall migration departure for adults versus juveniles, and tested if adults departed sooner after sunset, had greater flight speed of the first migratory flight, and a higher overall migration pace south through the eastern U.S. compared to juveniles. To compare flight speed for juveniles versus adults we used the first stage of migration which was the ~ 60 km crossing of Lake Erie where birds were detected departing from the north shore and arriving on the south shore the same night, allowing us to estimate flight speed of the first migratory flight. To compare the overall migration pace for juvenile and adult birds we measured the period of time that elapsed between detections for birds that were detected at multiple towers while migrating south through the eastern United States.

## Methods

### Study area

**Fig. 1:**
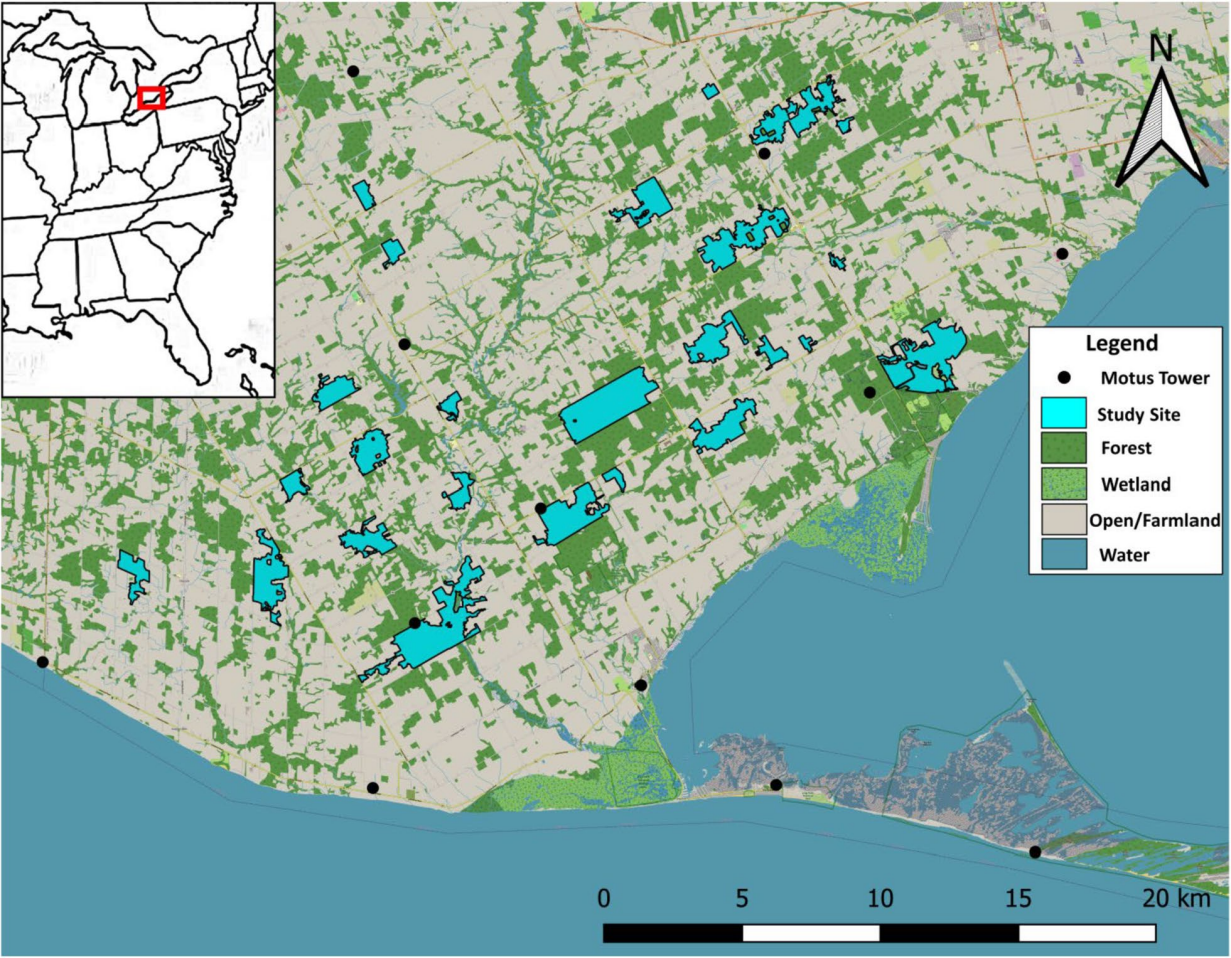
Map of the study area located in Norfolk County, Ontario, where adult and juvenile Wood Thrushes were tagged at 25 sites during the 2016–2019 breeding seasons

This study was conducted in Norfolk County, southwestern Ontario (42.6914 °N, 80.4877 °W), Canada (Fig. 1), which lies on the north shore of Lake Erie and has 21% forest cover that is composed of a wide variety of deciduous and mixed forest fragments of varying sizes [37]. Norfolk County has the most extensive Motus coverage in Ontario, with 13 towers resulting in a projected near-complete coverage for birds in migratory flight. During May–August from 2016 to 2019, nestling and adult Wood Thrushes were tagged in a total of 29 different forest fragments (16–499 ha in size) on a mix of public and private land that had similar forest structure and assemblages of dominant species of shrubs and trees [10, 28]. Dominant tree species consisted of maples (*Acer spp.*), oaks (*Quercus spp.*), White Pine (*Picea glauca*), American Beech (*Fagus grandifolia*), and Yellow Birch (*Betula alleghaniensis*), while dominant shrub species consisted of Spicebush (*Lindera benzoin*), Mapleleaf Viburnum (*Viburnum acerifolium*), American Witch-hazel (*Hamamelis virginiana*), Beaked Hazelnut (*Corylus cornuta*), and Chokecherry (*Prunus virginiana*). Necessary permits were obtained from the Ontario Ministry of Natural Resources and Forestry, Nature Conservancy Canada, Long Point Region Conservation Authority, and Long Point Basin Land Trust for conducting research on public lands, and landowner permission was sought during each spring for access to private lands.

### 2.2 Study species

Wood Thrushes are large-bodied enough to carry a radio transmitter with a year-long battery life, and previous studies found no negative effects of radio transmitters on survival and behavior [24]. Wood Thrushes have been previously tracked year-round using similarly-sized archival light level geolocators [63, 65, 66]. Their nests are often easy to find and monitor and many females produce multiple broods [26]. Wood Thrushes are a federally listed Threatened Species at Risk in Canada but are still a common breeding species in the deciduous and mixed forest fragments of southwestern Ontario [18]. In recent decades Wood Thrushes have declined across most of their breeding range due to habitat loss and fragmentation (e.g. Taylor and Stutchbury [67]).

### Nest searching, monitoring, and radio-tagging

Beginning on May 15 each year, all the study sites were searched for nests starting at the location of each singing male. Nests were checked every 6–8 days using a cellphone attached to a pole to record a video of the nest contents that could be reviewed away from the nest. When nestlings were observed, age was estimated by assessing feather growth. When nestlings were 10 days old, shortly before fledging which occurs at 12–14 days, they were removed from the nest and banded with both Canadian Wildlife Service numbered aluminum bands and unique color band combinations. The largest nestling (by mass) had a blood sample (< 25 uL) taken for genetic sexing (HealthGene Molecular Diagnostic and Research Center, Concord, Canada) and was equipped with a uniquely coded radio transmitter using a backpack leg loop harness [53] made from 2.5 mm Teflon tubing. Only one nestling was tagged at most nests (82%; 131 of 160 nests) but two nestlings were tagged at 29 nests that fledged late in the season. Of all tagged nestlings (n = 189), 133 survived to 16 days post-fledging and 119 survived to the onset of fall migration [28]. We refer to this age class as juvenile (e.g. Mitchell et al. [42], Patchett et al. [50]), although other studies on age-related migration timing use the terms hatch year [12, 14] or young [45].

Adults were caught by placing mist nets in the vicinity of nests and then banded with both Canadian Wildlife Service numbered aluminum bands and unique color band combinations. As with the nestlings, breeding adults (n = 117; 32 male and 85 female) were equipped with a uniquely coded radio transmitter using a backpack leg loop harness [53] made from 2.5 mm Teflon tubing. Sex was determined by checking for the presence of a brood patch or cloacal protuberance and age was determined as either second year (i.e., first breeding season) or after second year using plumage criteria [51].

The specifications of the radio-tags varied among years (2016: NTQBW-6-1 (1.6 g); 2017: ANTC-M6-1 (1.7 g); 2018: ANTC-M6-1 and NTQB2-6-1 (1.6 g); 2019: NTQB2-4-2S (1.5 g)) due to the manufacturer (Lotek Wireless Inc., Newmarket, Canada) discontinuing models, but all had similar performance, a burst rate of 12.7 s, and minimum expected lifespan of 400 days.

### Motus migration detections

The Motus Wildlife Tracking System is an automated radio telemetry system that is able to detect radio-tags remotely and thereby record individual bird movement over a regional scale with high temporal resolution, making it ideal for this type of study [68]. Each Motus Nanotag transmits a unique coded signal on the same frequency, so a single receiver is capable of detecting all tags in the network [68]. Using Motus eliminates one of the main problems associated with archival GPS and light-level geolocators: the need to recover the tags to retrieve the data. With Motus, the timing of onset of fall migration can be determined precisely, even for birds that are never seen again. Motus can also determine onset of fall migration when it occurs in mid-late September whereas light-level geolocators do not accurately estimate latitude for movements that occur close to the autumn equinox [11, 65].

Motus receivers sometimes record false detections due to random radio noise, duplicate tags, and overlapping tag signals when multiple tags are transmitting in the same area [19]. A number of filtering and quality control steps were taken to identify these and exclude them from analysis. First, we eliminated detections of fewer than 3 consecutive tag bursts because they are likely to be false detections [19]. Next, we eliminated any detection that occurred in impossible locations based on prior knowledge of migration timing and routes from geolocator studies [63, 65].

Detections representing the initiation of fall migration were those that occurred after sunset during the migratory period (August 25–October 15), when a bird was not detected in Ontario again until the following year. We used the time stamp of the first detection on the migration date to represent the time of day of the start of migration. Because the timing of sunset (the moment the sun disappears below the horizon) varies greatly during the migratory period, we calculated the number of minutes after sunset each bird began its migration at and used this number as our response variable. Motus towers cannot determine when a bird actually begins a migratory flight, only when it is first detected near the origin site. For this reason, we removed one extreme outlier from analysis, an adult that was detected 84 min later than the next latest bird, more than 5 h after sunset.

Our study area lies on the north shore of Lake Erie which gave us the unique opportunity to estimate flight speed of the initial migratory flight of birds that were detected departing the study area and subsequently detected along the south shore of Lake Erie. We cannot assume that the movements between stations are linear, but we can be reasonably certain that birds did not land in the water between detection at these towers. Signal strength readings when a bird flies past a tower create a parabolic arc with increasing strength heading toward the tower and decreasing strength after passing the tower. Because there is variation in the range at which a tower can detect a bird, we calculated the amount of time it took a bird to fly from peak signal strength at the last tower on the north shore of Lake Erie to the peak signal strength at the first tower on the south shore of Lake Erie [8], Additional Fig. 1). We measured the distance between the two towers and using the time elapsed between peak detections, calculated the average flight speed (km/h) and used this as our response variable. Some birds were detected before and after crossing Lake Erie, but were not picked up by one or both towers long enough to show the expected rising and falling of signal strength. These individuals (n = 11 adults; 8 juveniles) were removed from analysis because without a clear peak in signal strength, we could not accurately estimate the time when they passed each tower.

The direction and speed of wind was found to be an important predictor of migratory flight speed [43] so we included it as a covariate in our flight speed models. Following the methods of Morbey et al. [44], we used the RNCEP package [36] in R to extract easterly (90°) and northerly (0°) wind components at the 925 mb pressure level, which corresponds to an altitude of 675–825 m over Lake Erie and is comparable to the altitude of migratory flights measured in other species of thrushes [9]. We used linear interpolation with the *NCEP.interpol* function to interpolate 925 mb wind conditions at the time and date of departure for each bird. To estimate the tailwind component, we used *Vw* × cos(β), where *Vw* is the wind speed and β is the difference between the flight bearing and wind direction [56]. We assumed a flight bearing of 180°, as most birds were detected almost directly south of the study area after crossing the lake. This resulted in a tailwind metric that ranged from about − 10 when flying directly into a strong headwind, to about 10 when benefitting from a strong and direct tailwind.

The last measure we examined was the pace of migration through the U.S. after the night of the initial migratory flight. Motus does not allow us to determine when a bird is stopping over (e.g., relatively stationary for one or more nights) versus migrating because of gaps in tower coverage, but we can measure the time it takes a bird to travel between towers and measure the distance between those towers to calculate the average number of kilometers a bird travels per day. If a bird is spending more time stopping over, it will travel fewer kilometers per day when covering the distance between towers. To ensure that we were measuring stopover behavior and not speed of direct overnight flights, we eliminated detections that occurred on consecutive days (n = 2). We detected 25 individuals at multiple towers, not on successive days, as they migrated south through the United States (Additional Fig. 2). In Swainson’s Thrushes (*Catharus ustulatus*) migration pace was found to be slower at higher latitudes [8], so we calculated the midpoint in latitude between Motus towers to include as a variable in analysis. We also included the distance between Motus towers because it was also found to be important to predicting migration pace in Swainson’s Thrushes, with a faster pace when detections were geographically closer to each other [8].

**Fig. 2:**
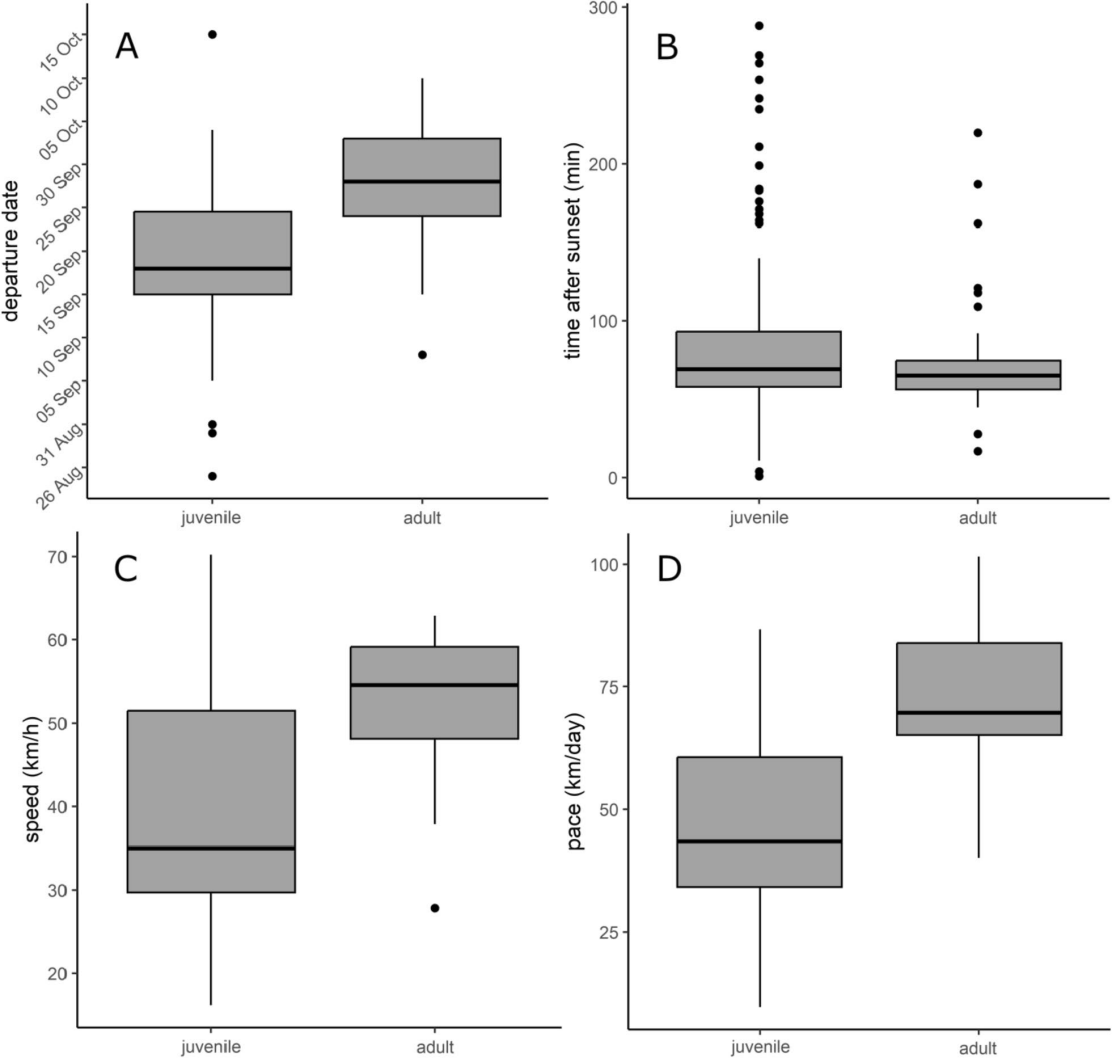
During 2016–2019, juvenile Wood Thrushes tagged in southwestern Ontario averaged earlier fall migration departure dates than adults (**A**), and the time of night they initiated migration was later than adults (**B**). Juvenile Wood Thrushes averaged a slower flight speed on their first migratory flight (**C**), and migration pace southward through the eastern U.S. was also slower than adults (**D**)

### Statistical analysis

All analyses were conducted in R 4.3.2 [52] and all tests were two-tailed and values are expressed as means ± SE. All scatterplots were created using the *ggplot2* package [71] in R. We used the *dredge* function in the *MuMIn* package [6] to rank candidate models using Akaike Information Criterion scores corrected for small sample size (AIC_c_). We considered models to be supported when their ΔAIC_c_ = 0–2 (the top model ΔAIC_c_ = 0) and at least 2 AIC_c_ better than the intercept-only model [13], but examined each supported model to avoid assigning importance to uninformative parameters [5]. To evaluate predictor variables in each supported set of models, we used model averaging and calculated 95% confidence intervals. For linear models we examined residual plots to check model assumptions and for generalized linear models we used the DHARMa package [27] to standardize residuals and plot them against rank-transformed model predictions to check model assumptions.

To test whether fall migration initiation date, flight speed, and migration pace differed between adult and juvenile Wood Thrushes, we fit linear models using age (adult or juvenile) as our predictor and included the covariates sex and year using the *stats* package (R Core Team [52]). For our fall migration initiation date analysis, we also included an interaction between age and sex because adult female Wood Thrushes initiate fall migration an average of five days earlier than males [10], while juvenile female Wood Thrushes average only slightly earlier migration initiation than males [28]. For our migration pace analysis, we used linear mixed models and included individual as a random effect using the R package *lme4* [7] to account for some individuals with data for multiple legs of the same migration (n = 4). For this analysis, we included the latitude halfway between detections and number of days between detections, to see if pace varies by latitude and the length of time between detections. We also included the date of migration initiation to see if late departing birds compensate with a faster pace. To test whether the time of day that Wood Thrushes departed for migration differed between adults and juveniles, we fit generalized linear models with a Gamma error distribution and logarithmic link using age (adult or juvenile) as our predictor and included the covariates sex and year.  

## Results

### Fall migration initiation date and time of night

We deployed tags on 117 adults and 189 nestling Wood Thrushes, with 119 of these nestlings surviving both the fledgling and pre-migration period [28]. Of these birds, we detected 60/117 (51%) adults (19 male, 41 female) and 82/119 (69%) juveniles (43 male, 39 female) departing the study area as they initiated fall migration. The earliest migratory flight occurred on Aug 25, and the latest was October 15 with most departures occurring in mid to late September for both age classes (juveniles: Sept 19 ± 0.9 days; adults: Sept 28 ± 0.7 days). Juveniles departed significantly earlier than adults (*t(139)* = *− 7.99, P* < *0.001)* and the effect size was 9 days (Fig. 2A). The three supported models (Table 1) were age + sex + year + age*sex (*w*_i_ = 0.35), age + sex + age*sex (*w*_i_ = 0.30), and age + sex (*w*_i_ = 0.16). Age had a positive relationship with the migration departure date in the three supported models and the 95% confidence interval did not include zero (Table 2). Sex, year, and an interaction between sex and age also appeared in the supported models having a positive relationship with migration initiation date; however, the effect was weak because the 95% confidence intervals included zero.

**Table 1:** Models with ΔAIC_c_ < 2 that are at least 2 AIC_c_ better than the intercept-only model for each response variable of interest

Response variable	Model	LL	*n*	*k*	AIC_c_	ΔAIC_c_	*w*_i_
Migration date	Age + sex + year + age*sex	− 474.2	142	6	961.1	0.0	0.35
	Age + sex + age*sex	− 475.5	142	5	961.4	0.28	0.30
	Age + sex	− 477.2	142	4	962.7	1.61	0.16
Time of day	Age	− 727.1	141	3	1460.3	0.0	0.29
	Age + year	− 726.1	141	4	1460.4	0.14	0.27
	Age + year + sex	− 725.5	141	5	1461.5	1.14	0.16
	Age + sex	− 726.7	141	4	1461.8	1.47	0.14
Speed (km/h)	Tailwind + year	− 99.1	27	4	208.1	0.0	0.33
	Tailwind	− 98.7	27	3	209.4	1.35	0.17
	Age + tailwind	− 99.2	27	4	209.6	1.50	0.16
Pace (km/day)	Age + sex + year + latitude	− 119.6	31	7	258.0	0.0	0.28
	Age + sex + year + latitude + departure date	− 118.1	31	8	258.7	0.74	0.19

All models with *w*_i_ ≥ 0.05 for each analysis available in additional files (Additional Table 1). All data was collected during 2016–2019 as birds departed from the study site in Norfolk County, Ontario, or as they travelled south through the eastern United States

**Table 2:** 95% confidence intervals for averaged coefficient estimates appearing in models with ΔAIC_c_ < 2 that are at least 2 AIC_c_ better than the intercept-only model for each response variable of interest

Response variable	Coefficient	Estimate	2.5%	97.5%
Migration date	Intercept	− 770.7	−3839.0	2297.6
	Age	8.2	4.7	11.6
	Sex	1.1	−2.1	4.3
	Year	1.2	−0.33	2.7
	Age*sex	4.9	−0.026	9.9
Time of day	Intercept	89.3	−155.1	333.7
	Age	− 0.24	−0.44	− 0.03
	Sex	− 0.09	−0.30	0.11
	Year	− 0.08	−0.21	0.042
Speed (km/h)	Intercept	− 7353.4	− 25,319.1	10,612.30
	Age	6.8	− 2.2	15.8
	Year	7.3	− 0.27	14.80
	Tailwind	2.3	1.3	3.3
Pace (km/day)	Intercept	1665.8	− 35,961.9	39,293.6
	Age	19.0	− 0.30	38.3
	Sex	3.3	− 12.6	19.2
	Year	− 0.8	− 19.4	17.8
	Latitude	− 3.3	− 6.0	− 0.66
	Departure date	0.99	− 0.27	2.3

All data was collected during 2016–2019 as birds departed from the study site in Norfolk County, Ontario, or as they travelled south through the eastern United States

We determined time of day for the initiation of migratory flight for 59 adults, and 82 juvenile birds. All Wood Thrushes initiated migratory flight between 1 and 288 min after sunset with the average being 84 ± 4 min after sunset. We found a significant difference in the time of day birds began migration based on their age (*t(128)* = *2.51, P* = *0.01*), with adults starting migration an average of 72 ± 4 min after sunset and juveniles starting migration an average of 93 ± 7 min after sunset (Fig. 2B). The four supported models (Table 1) were age (*w*_i_ = 0.29), age + year (*w*_i_ = 0.27), age + year + sex (*w*_i_ = 0.16), and age + sex (*w*_i_ = 0.14). Age had a negative relationship with timing of the initial migratory flight in the four supported models and the 95% confidence interval did not include zero (Table 2). Sex and year also had a negative relationship with the timing of the initial migratory flight, but in both cases the 95% confidence interval included zero.

### First migratory flight speed and migration pace

**Fig. 3:**
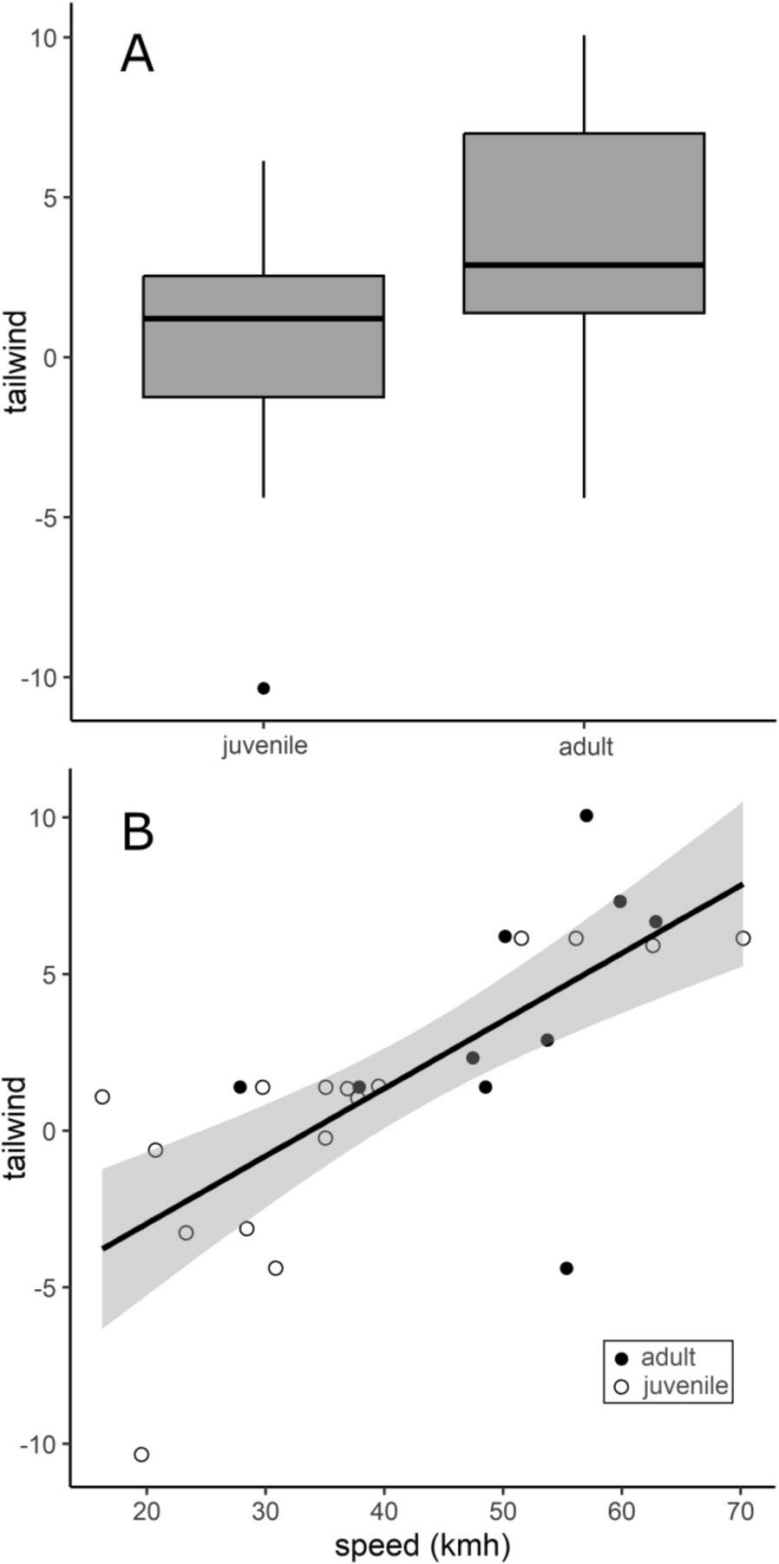
During 2016–2019 fall migration in Norfolk County, Ontario, adults averaged a greater wind advantage during their flights across Lake Erie than juveniles (**A**). The advantage gained from tailwind was closely linked to flight speed, and adults were more likely to initiate migration on nights with favorable wind conditions (**B**). The tailwind metric ranges between about 10 and −10 and indicates the relative advantage the wind gives a bird during flight. Wind speed and direction are used to calculate the tailwind metric, with a direct and strong tailwind producing higher positive scores and a direct and strong headwind producing lower negative scores

After filtering all detections, we ended up with 16 juvenile and 11 adult Wood Thrushes that were detected on both sides of Lake Erie with clear peaks in signal strength at both towers. Calculated flight speed of birds crossing the lake varied from 16.3 to 70.2 km/h and the average flight speed was 42.7 ± 2.9 km/h. When not accounting for other variables, we found a significant difference in the flight speed of adult and juvenile birds (*t(25)* = − *2.76, P* = *0.003*), with juveniles (37.1 ± 3.9 km/h) averaging about 25% slower than adults (51.0 ± 3.1 km/h; Fig. 2C). However, adults had a stronger tailwind component than juveniles (Fig. 3A) and thus in the overall model, age class was not a strong predictor of flight speed (Table 1). The three supported models (Table 1) were tailwind + year (*w*_i_ = 0.33), tailwind (*w*_i_ = 0.17), and tailwind + age (*w*_i_ = 0.16). Age class appeared in the supported models with a positive relationship with flight speed, but the 95% confidence interval included zero indicating a weak relationship (Table 2). The tailwind component had a positive relationship with flight speed (Fig. 3B) and was the only covariate with a 95% confidence interval that did not include zero (Table 2).

Despite the low Motus coverage across the interior eastern U.S., we were able to determine the speed of 31 different sections (the linear distance between two towers that captured consecutive detections of the same bird) of migration by 25 individual birds. Of these, 13 birds were juvenile (n = 18 sections), and 12 were adults (n = 13 sections). Migration pace varied from 9.8 to 101.9 km/day and the average pace was 57.4 ± 4.1 km/day. Migration pace differed significantly by age (*t(29)* = *− 3.49, P* = *0.002)*, with juvenile birds (47.3 ± 5.1 km/day) averaging a 34% slower pace than adults (71.6 ± 4.7 km/day; Fig. 2D). The two supported models (Table 1) were age + sex + year + latitude (*w*_i_ = 0.28), and age + sex + year + latitude + distance (*w*_i_ = 0.18). Age appeared in both models having a positive relationship and a 95% confidence interval that narrowly included zero (Table 2). Sex and departure date were positively related to migration pace and had 95% confidence intervals that included zero. Latitude had a negative relationship with migration pace, with pace increasing farther south, and the 95% confidence interval did not include zero.

## Discussion

We used Motus to measure and compare different migration parameters of juvenile and adult Wood Thrushes departing from a breeding site, giving us the unique opportunity to test predictions based on different age classes starting migration from the same location. We found that adults departed on fall migration at a later date, sooner after sunset, and had a faster migration pace through the United States. We also found that adults crossed Lake Erie faster during their initial migratory flight, but the speed and direction of tailwind on the night of departure was the strongest contributing factor.

### Migration timing

Although many studies have tested for age differences in migration timing at stopover sites (e.g. Carlisle et al. [14]), few have tested for differences in the initial onset of fall migration. We found that juvenile Wood Thrushes initiated fall migration at a significantly earlier date than adults, by 9 days on average. Savannah Sparrow juveniles started their fall migration 3–4 weeks earlier than adults [20, 31] but juvenile Cyprus Wheatears (*Oenanthe cypriaca*) depart their natal site on fall migration 5 days later than adult breeders [50]. The later start of adult migration in some species may be due to time/energy constraints arising from the timing of feather molt, breeding, and parental care (e.g. Mitchell et al. [42]). At migratory stopover sites, a strong pattern across passerine species is that juvenile fall migration precedes that of adults if adults undergo extensive feather molt prior to migration whereas juveniles migrate later than adults for species in which adults undergo molt during or after migration (e.g. Carlisle et al. [14]). The Wood Thrush fits this pattern since adults molt ~ 90% of their flight feathers at the breeding site prior to migration [25] and late breeding adults delay molt and fall migration presumably to avoid overlap in these energetically costly activities [65]. Many other studies on migratory passerines have shown that within populations late breeding adults delay departure on fall migration [15, 20, 21, 33, 42]. Juvenile Wood Thrushes do not face as strong time constraints in part because they do not molt their flight feathers until the next year (e.g. Evans et al. [23]). However, Wood Thrushes that fledged very late in the season (late July–early August) began fall migration about 10 days later than the earliest fledged birds (mid-June, [28]). For late fledged juveniles, there may be a tradeoff between taking more time to prepare for their first migration (e.g., to be in better body condition) versus the costs of later departure (e.g., decreasing temperature and food resources; Mitchell et al. [42]).

Initiating migratory flights soon after sunset is thought to be a strategy that maximizes the potential for long distance nocturnal flights [17, 47]. Later departure after sunset of juveniles versus adults is hypothesized to be due to their inexperience in using orientation cues and assessing favorable environmental conditions for long flights [57]. Juvenile Wood Thrushes departed on their first migration flight significantly later after sunset than adults, but only by 21 min on average (29% later). Few studies have tracked the time of night of the first fall migration flight of juvenile and adult passerines from the same breeding site [17]. For Kirtland’s Warbler (*Setophaga kirtlandii*) there was no significant difference in nocturnal departure time from the natal/breeding site for juveniles versus adults (both ~ 50 min after sunset [17]. It is unclear if the later nocturnal departure by juvenile Wood Thrushes is related to their inexperience and/or if juveniles have a shorter first migration flight than adults and so are not as time constrained. We also cannot confirm if this pattern continues after the first migratory flight, and whether the difference would be biologically meaningful during a long migration. Juveniles and adults crossed the ~ 60 km water barrier of Lake Erie on their first fall migration flight, but we do not know how much farther they travelled on that night or whether sunrise was the limiting factor of the flight duration.

Some passerines that have been tracked leaving their breeding/natal site undergo a period of short distance regional movements prior to onset of long-distance fall migration (Brown and Taylor [12], reviewed by Züst et al. [74]). For juveniles, these movements may function to increase access to food, to search for future breeding territories, to create a homing target for spring migration, and/or to train the stellar compass and orientation in preparation for migration (e.g. Mukhin et al. [46], Mitchell et al. [41]). Across passerines, these pre-migratory regional movements typically occur two hours later after sunset than migration departure flights perhaps because they are shorter distance than migration movements (e.g. Cooper et al. [17]). For our population of Wood Thrushes, adults rarely (8%) made regional movements before the onset of fall migration, but most juveniles (78%) made regional movements of up to 30 km away from the natal site [29]. Juvenile pre-migratory movements occurred primarily during the two weeks before fall migration departure, during the two hours prior to sunrise and were random in direction [29]. While most juveniles had gained experience travelling > 5 km in darkness prior to fall migration they may still have been inexperienced in orientating appropriately for longer distance southward migration. If later departure after sunset is due to inexperience, the age difference should diminish as the journey progresses. However, in the Northern Wheatear subspecies (*Oenanthe oenanthe leucorhoa*) that breeds in Canada, Greenland and Iceland, radio-tracking at an island stopover site in Germany showed that juveniles that were already > 800 km from natal sites (and so were experienced with long overwater flights) still departed at night about 80 min later than adults [57]. Further tracking studies from breeding sites are needed to understand the ecological factors, and biological significance, of age-related differences nocturnal departures on migration flights.

### Flight speed and migration pace

On their first migratory flight, juvenile Wood Thrushes averaged 25% slower flight speed than adults, but this was due to adults using tailwind more so than juveniles. Tailwind was a strong predictor of flight speed, but age class was not. Similarly, in Savannah Sparrows slower inaugural migration flight in juveniles from an island was due to juveniles departing under less beneficial wind conditions [43]. Cyprus Wheatear fall migration starts with a very long (> 1000 km) non-stop flight, yet juvenile migration speed was not slower than adults perhaps because of the consistent availability of tailwinds [50]. Juveniles may learn to use tailwinds once fall migration is underway [57, 62]. Deppe et al. [22] used automated telemetry to track fall migration of individual Swainson’s and Wood Thrushes departing the U.S. Gulf coast and arriving in the Yucatan Peninsula after crossing across the Gulf of Mexico. Age class had no effect on individual departure decisions which were associated with passage of a cold front (providing tail winds) and high fat reserves, and juveniles did not take longer than adults to fly the ~ 1000 km non-stop journey which for Wood Thrushes lasted an average of 28 h.

We found that juvenile Wood Thrushes averaged a 34% slower pace than adults as they moved south through the eastern U.S. In our study population, juvenile fall migration occurs almost two months, on average, after young become independent of their parents [28]. While juveniles have much experience with foraging prior to fall migration, refueling at stopover sites may be more challenging because it requires physiological and behavioral changes to promote high food intake and fat storage and doing so at unfamiliar locations [38]. In Blackpoll Warblers (*Setophaga striata*) juveniles preceded adults by about one week at stopover sites in the western U.S. but this age pattern was reversed at eastern stopover sites presumably because juveniles migrate across North America more slowly than adults [45]. Juveniles are often hypothesized to face proximate constraints on migration pace due to their inexperience, such as inefficient foraging, which should be especially so during fall migration (reviewed by Woodrey [70]). Many studies have documented a lower body mass of juveniles than adults at stopover sites, which has been assumed to reflect differences in refueling ability (reviewed by Woodrey [70]). However, Jones et al. [34] examined passerine recaptures during fall stopover in southwestern Ontario to estimate rate of daily mass gain and found only 2 of 47 species had lower mass gain in juveniles than adults (89% of species had no significant age difference). Seewagen et al. [58] used plasma metabolite analysis to quantify refueling rate at a fall stopover site in New York and found no significant differences between juveniles and adults in both Neotropical migrants studied (Swainson’s Thrush and Common Yellowthroat *Geothlypis trichas*). Although juveniles are relatively inexperienced, they have evolved a suite of physiological adaptations in response to the daunting energetic challenges of their first migration (e.g. McCabe and Guglielmo [38]). Juvenile passerines may be able refuel efficiently because they have larger digestive organs, higher metabolic rates, and higher rates of food intake compared with adults [38].

Fall migration pace is influenced by proximate factors along the journey, such as food supply and environmental conditions, but also by the longer-term selection pressures that influence migration strategy (e.g. Hedenstrom [3], Alerstam [30]). Selection pressures act on increasing immediate migration survival (e.g., time/energy optimization and predator avoidance) but may also occur through carry-over effects of migration pace on subsequent stages of the annual cycle (e.g. Marra et al. [40]). Many migratory passerines defend individual winter territories, and studies have shown that a high-quality winter territory can improve late-winter body condition, advance the timing of spring migration and increase reproductive success through early arrival on the breeding grounds (e.g. Norris et al. [48], Reudink et al. [54]). However, early spring arrival may not be as beneficial for juveniles because of the costs of competing with returning adults for breeding territories. McKinnon et al. [39] tracked spring migration of juvenile and adult Wood Thrushes from wintering sites in Belize and Costa Rica and found that the duration of spring migration of juveniles was 50% longer than adults even though juveniles took a similar route across the Gulf of Mexico. Juveniles made more frequent and shorter stopovers as they moved farther north through the U.S. which may be a stalling strategy rather than a pace imposed by flight inefficiency or slow refueling. For fall migration timing, it is unclear whether juveniles and adults face strong carryover effects that will influence their winter body condition or survival. We do not know if juvenile Wood Thrushes arrive on the wintering grounds later than adults or if this affects their access to high quality winter resources. We suspect not, because wintering juveniles do not consistently have lower body condition than adults [39, 64], the winter home ranges of juveniles do not contain a lower availability of food [64], and the timing of fall migration initiation in juveniles does not predict apparent annual survival [28]. Wood Thrushes also do not defend a fixed territory site throughout the wintering season and most individuals (~ 70%) move regionally (1–148 km away) long before spring migration begins [64].

## Conclusions

The potential carry-over link between early fall migration departure and increased over-winter fitness is weakened by the discovery that many passerines have prolonged (> 7 d) fall stopovers, which are considered longer than necessary to refuel for migration [4]. Half of adult Wood Thrushes tracked with geolocators had prolonged stopovers on fall migration, and therefore the timing of fall migration through the southern U.S. did not predict arrival date on the wintering grounds [65]. Automated telemetry has confirmed that juveniles of some species (Blackpoll Warbler, Red-eyed Vireo) also have prolonged stopovers on fall migration [61]. A prolonged fall stopover can be associated with the need to cross large water bodies like the Gulf of Mexico but these also occur in other landscape contexts and so the fitness consequences are not well understood.

We found evidence that juvenile Wood Thrushes have an earlier and slower fall migration than adults. Juveniles departed at an earlier date, had a later nocturnal migration departure time from the natal/breeding site than adults, were less likely to use tailwinds on their first migration flight across a water barrier, and had a slower pace of fall migration through the eastern U.S. However, distinguishing between the proximate (e.g. wing length, experience, physiology) and ultimate (e.g. fitness benefits of earlier or faster fall migration) mechanisms causing these age-related patterns remains a challenge. More tracking studies from breeding sites are needed to understand the age-related differences in migration performance and we suggest comparing species with/without winter territories and with/without prolonged fall stopovers as a practical starting point.

## Supplementary Information

Additional file 1.

## Data Availability

The datasets used for analysis in this study have been uploaded to Dryad will be publicly available with a doi after review. Reviewers link: http://datadryad.org/stash/share/sLOL4mXxruXsinAr41y41is_-flkef3XtGuy8HFrACg.
