# Supplementary material for: Age-related differences in fall migration timing and performance of juvenile and adult Wood Thrushes departing from a breeding site
Source: Mov Ecol. 2025 May 6;13:32. doi: 10.1186/s40462-025-00556-3 (PMC12057278; doi:10.1186/s40462-025-00556-3)
Supplement: Supplementary file 1 — Additional file 1. [file 40462_2025_556_MOESM1_ESM.docx]

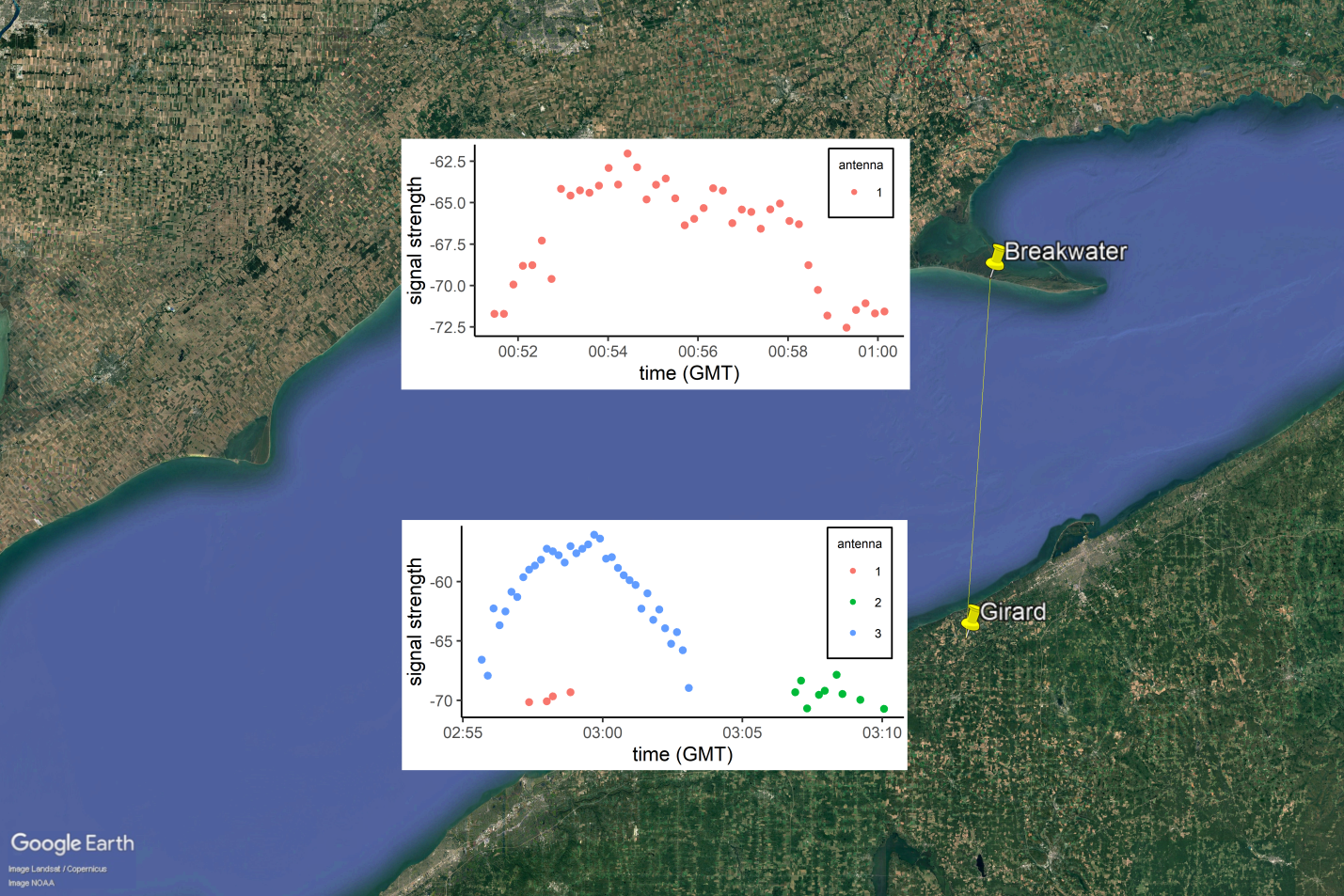


**Additional Figure 1.** When crossing Lake Erie during migration on the evening of Sept 25 2017, juvenile Wood Thrush #381 passed Breakwater tower with a signal strength peak at 00:54:25 GMT and was then detected passing Girard tower just over two hours later with signal strength peaking at 02:59:41 GMT. The straight line distance of 64.1km was covered at an average speed of only 30.83kmh which is consistent with the tailwind metric of -4.38 during the crossing.

**
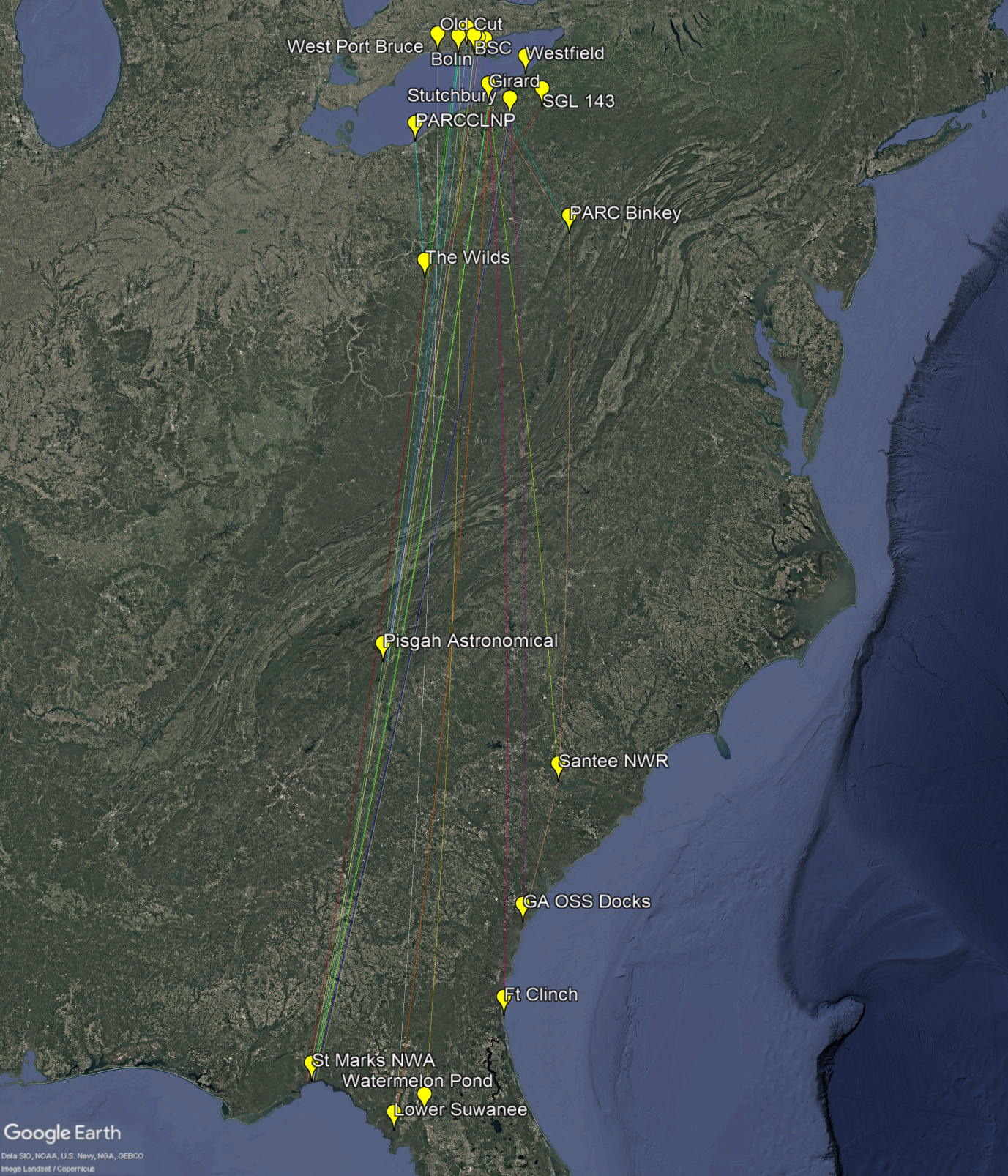
**

**Additional Figure 2.** As tagged Wood Thrushes migrated south through the eastern United States during fall in 2016-2019, they were detected by Motus receivers along the route. Each of the 25 individuals that were detected at multiple receivers is represented by a unique color and in total we recorded 29 segments that included multiple segments from four birds.

**Additional Table 1.** All models with *w*_i_ ≥ 0.05 and ΔAIC_c_ <2 that are at least 2AIC_c_ better than the intercept-only model for each response variable of interest.

| response variable | **model** | **LL** | ***n*** | ***k*** | **AIC_c_** | **ΔAIC_c_** | ***w*_i_** |
| --- | --- | --- | --- | --- | --- | --- | --- |
| migration date | age + sex + year + age*sex | -474.2 | 142 | 6 | 961.1 | 0.0 | 0.35 |
|  | age + sex + age*sex | -475.5 | 142 | 5 | 961.4 | 0.28 | 0.30 |
|  | age + sex | -477.2 | 142 | 4 | 962.7 | 1.61 | 0.16 |
|  | age + sex + year | -476.5 | 142 | 5 | 963.4 | 2.32 | 0.11 |
|  | age | -479.3 | 142 | 3 | 964.9 | 3.77 | 0.05 |
| time of day | age | -727.1 | 141 | 3 | 1460.3 | 0.0 | 0.29 |
|  | age + year | -726.1 | 141 | 4 | 1460.4 | 0.14 | 0.27 |
|  | age + year + sex | -725.5 | 141 | 5 | 1461.5 | 1.14 | 0.16 |
|  | age + sex | -726.7 | 141 | 4 | 1461.8 | 1.47 | 0.14 |
|  | year | -728.4 | 141 | 3 | 1463.1 | 2.76 | 0.07 |
| speed (km/h) | tailwind + year | -99.1 | 27 | 4 | 208.1 | 0.0 | 0.33 |
|  | tailwind | -98.7 | 27 | 3 | 209.4 | 1.35 | 0.17 |
|  | age + tailwind | -99.2 | 27 | 4 | 209.6 | 1.50 | 0.16 |
|  | age + tailwind + year | -98.6 | 27 | 5 | 210.1 | 2.00 | 0.12 |
|  | tailwind + sex + year | -98.7 | 27 | 5 | 210.3 | 2.25 | 0.11 |
| pace (km/day) | age + sex + year + latitude | -119.6 | 31 | 7 | 258.0 | 0.0 | 0.28 |
|  | age + sex + year + latitude + departure date | -118.1 | 31 | 8 | 258.7 | 0.74 | 0.19 |
|  | age + latitude + year | -122.6 | 31 | 6 | 260.8 | 2.78 | 0.07 |
|  | age + latitude + sex | -122.7 | 31 | 6 | 260.9 | 2.89 | 0.07 |
|  | age + latitude + year + departure date | -121.1 | 31 | 7 | 261.0 | 3.04 | 0.06 |
|  | age + latitude + sex + departure date | -121.2 | 31 | 7 | 261.2 | 3.23 | 0.06 |
